# Short-Term Effects on Tear Film Following Cosmetic Botulinum Toxin Injections in Healthy Individuals

**DOI:** 10.7759/cureus.77688

**Published:** 2025-01-19

**Authors:** Mübeccel Bulut, Ali Hakim Reyhan

**Affiliations:** 1 Ophthalmology, Necip Fazıl City Hospital, Kahramanmaraş, TUR; 2 Ophthalmology, Harran University, Şanlıurfa, TUR

**Keywords:** botulinum toxin-a (bont-a), ocular surface disease index (osdi), schirmer's test, tear break-up time (tbut), tear film

## Abstract

Purpose: Botulinum toxin-A (BoNT-A) injections have become increasingly popular for cosmetic treatments in the upper face region, particularly for reducing wrinkles and achieving aesthetic improvements. While the cosmetic benefits are well-documented, the potential effects of these injections on ocular surface health and tear film dynamics remain incompletely understood. This study aimed to comprehensively investigate the impact of BoNT-A injections on tear film parameters and ocular surface health across different time intervals following treatment.

Materials and methods: This cross-sectional study evaluated 80 participants divided into four groups of 20 each. Three groups consisted of patients who had received upper face BoNT-A injections within the previous six months, evaluated at different time intervals: group 1 (0-1 month post-injection), group 2 (1-3 months post-injection), and group 3 (3-6 months post-injection). The fourth group served as an age- and gender-matched control group with no history of BoNT-A treatment. A comprehensive assessment of ocular surface health was conducted using three standardized parameters: the Schirmer test (without anesthesia) for tear production, the tear break-up time (TBUT) for tear film stability, and the Ocular Surface Disease Index (OSDI) for symptom evaluation. All participants underwent standardized BoNT-A injection protocols, receiving a total of 50 units administered by the same experienced cosmetic surgeon.

Results: Significant differences in tear production were observed, with the 0-1 month post-injection group exhibiting the highest mean Schirmer score (18.35±7.63 mm), compared to 12.9±3.64 mm in the control group (p<0.01). TBUT measurements revealed greater tear film stability in the control (11.35±2.92 seconds) and 0-1 month (11.05±2.97 seconds) groups compared to the 1-3 and 3-6 month groups (p<0.05). OSDI scores indicated increased symptom severity in the 1-3 month (18.62±7.04) and 3-6 month (18.82±6.08) post-injection groups compared to the control group (11.82±2.67) (p<0.001).

Conclusions: Cosmetic BoNT-A injections can lead to alterations in tear film dynamics and ocular surface health, with effects varying across different post-injection time periods. The findings suggest an initial increase in tear production followed by potential alterations in tear film stability and increased dry eye symptoms in the months following treatment. These effects appear to vary over time following injection. Further research is now needed to fully elucidate the long-term impact of cosmetic BoNT-A injections on the ocular surface.

## Introduction

Botulinum toxin, a potent neurotoxin produced by the bacterium *Clostridium botulinum*, is known to paralyze the skeletal muscle by inhibiting the release of acetylcholine [1,2]. Seven distinct botulinum toxin types have been identified [3]. Botulinum toxin-A (BoNT-A) is regarded as a significant treatment modality in the field of facial rejuvenation [4]. BoNT-A has been shown to exhibit therapeutic effects for a variety of conditions, including strabismus, hyperhidrosis, dystonia, spasticity, overactive bladder, and migraines, providing relief and improving patients' quality of life [5]. The mechanism of action involves the inhibition of the release of acetylcholine at the neuromuscular junction, causing a loss in cholinergic signaling and resulting in the temporary denervation and modulation of muscle activity. However, acetylcholine also acts on sympathetic nerves in glandular tissues, where it stimulates the sweat and salivary glands [6].

Considering the widespread use of BoNT-A in cosmetic applications, it is crucial to exercise caution, particularly in high-risk patient groups. When administering BoNT-A injections in the periocular region, special attention should be given to the following patient groups: those with pre-existing dry eye disease, contact lens users, and individuals with other ocular surface disorders. Understanding these effects is essential for developing appropriate pre-treatment screening protocols, establishing post-treatment monitoring guidelines, and identifying patients who may require additional protective measures.

BoNT exerts biological and non-muscular effects on the human skin and other tissues. Epidermal keratinocytes, mesenchymal stem cells from the subcutaneous adipose tissue, nasal mucosal cells, urothelial cells, intestinal, prostate, and alveolar epithelial cells, neutrophils, and macrophages express the BoNT-A-binding proteins SV2, FGFR3 or vanilloid receptors, and/or the BoNT-A cleavage target SNAP-25 and SNAP-23. BoNT-A can also induce specific biological effects in dermal fibroblasts, mast cells, sebocytes, and vascular endothelial cells [7].

Botulinum toxin injection is today the most widely performed cosmetic procedure worldwide. The glabella region, which houses the procerus, orbicularis oculi, depressor supercilii, and corrugator muscles, is one of the most frequently treated areas for the reduction of muscle activity. The periorbital area is another common site targeted for correction [8]. However, it can also produce undesirable effects attributable to the diffusion of the toxin to adjacent regions. The effect of BoNT-A can spread up to 3 cm transversely from the initial injection site.

Neurotoxin injection has been shown to improve dry eye symptoms in patients with blepharospasm [9]. This raises the possibility that cosmetic BoNT-A injections may also impact the tear film and ocular surface.

Published research concerning tear film changes following cosmetic BoNT-A injection is scarce. The aim of this study was to evaluate the impact of BoNT-A injections administered for cosmetic purposes on tear film parameters, including tear production, tear film stability, and subjective dry eye symptoms, at different time intervals post-injection. 

## Materials and methods

This study was designed to evaluate ocular surface parameters in three distinct patient groups and a control group, with all groups being individually matched for both age and gender. Eighty participants were enrolled, 20 in each of the four groups. The study included individuals who had received upper-face BoNT-A injections within the previous six months.

BoNT-A injection was administered at Necip Fazıl City Hospital by Dr. Bulut following a standardized protocol. The procedure was performed under sterile conditions, utilizing predetermined dosages and specific anatomical landmarks. Intramuscular injections of 0.1 mL Botox® (onabotulinumtoxin A, Allergan, Irvine, California, United States) solution were delivered to each injection point, with each participant receiving a total of 50 units. Injections targeted the orbicularis oculi, glabellar, frontalis, and procerus muscles. All applications were carried out by the same experienced cosmetic surgeon, thus ensuring reproducibility and standardization.

Patients in group 1 were evaluated 0-1 month after BoNT-A injection, those in group 2 were evaluated 1-3 months post-injection, and those in group 3 were evaluated 3-6 months after injection. The control group consisted of individuals with no history of BoNT-A injection.

Having an ocular surface disease, currently using topical medications, with previous histories of prior eyelid or facial surgeries, thyroid eye disease, active smoking history, and wearing contact lenses were determined as exclusion criteria. Patients who applied to our outpatient clinic between April 2024 and September 2024 and had upper-face Botox applied for aesthetic purposes were included in the study. Since the patients did not have the mentioned exclusion criteria, all of them were included. Ocular surface health was assessed using three basic parameters, the Schirmer test, the tear break-up time (TBUT), and the Ocular Surface Disease Index (OSDI). 

The study was approved by the Harran University Clinical Research Ethics Board, on March 18, 2024, under protocol number HRU/24.02.56. All participants provided written informed consent. This research was conducted in full compliance with the ethical principles outlined in the Declaration of Helsinki.

The Schirmer test (without topical anesthesia) was conducted by placing a test strip behind the lower eyelid, between the temporal and middle third. This was removed after five minutes, and the length of the wet portion was measured.

TBUT was measured by applying a drop of 0.9% sterile saline as a wetting agent to a fluorescein strip, which was then used to dye the ocular surface. This allowed the examiner to use a cobalt blue filter to view the cornea and measure the break-up time, defined as the time between the patient's last complete blink and the formation of the first black spot or line, indicating a break in the tear film. The test was performed in duplicate, with an average value being calculated. A reading of ≤10 seconds was considered abnormal.

The OSDI questionnaire, divided into three categories (symptoms of eye irritation, functional problems, and environmental triggers contributing to or causing ocular surface), consists of 12 questions, each rated on a five-point scale based on frequency: 0 for none, 1 for occasional, 2 for approximately half the time, 3 for most of the time, and 4 for continuous occurrence. The final OSDI score is calculated by summing the scores for the 12 questions, dividing this by the total number of questions answered, and then multiplying the result by 25. According to the Chinese Dry Eye Expert Consensus on Dry Eye Associated with Ocular Surgery, an OSDI score of 13 or greater indicates the presence of dry eye symptoms (see Appendices).

Statistical analyses

Statistical analyses were performed using R statistical software, Version 4.1.2 (R Foundation for Statistical Computing, Vienna, Austria). The normality of the continuous variables was not explicitly tested in this analysis, but this is generally assumed for ANOVA. The variables were analyzed and expressed as mean±standard deviation (though these specific descriptive statistics were not calculated in our analysis). The sample size was determined to detect a medium effect size with a significance level of 5% and a statistical power of 80%. All analyses were conducted with a 95% confidence interval. A p-value less than 0.05 was considered statistically significant for all analyses.

## Results

This study involved 160 eyes of 80 patients (60 (75%) female, 20 (25%) male), with a mean age of 42 years (range: 34-58 years; women: 41 years, men: 43 years), constituting a comprehensive sample for the evaluation of ocular surface parameters in relation to BoNT-A injections.

Schirmer test results revealed significant differences in tear production among the groups, with group 1 (0-1 month post-injection) registering the highest mean score (18.35±7.63 mm) and the control group the lowest (12.9±3.64 mm). The difference between group 1 and the control group was statistically significant (p<0.01).

TBUT measurements indicated variations in tear film stability. The control group and group 1 exhibited higher mean TBUT values (11.35±2.92 and 11.05±2.97 seconds, respectively) than groups 2 and 3. Significant differences were observed between groups 2 and 3 versus the control group (p<0.05).

**Figure 1:**
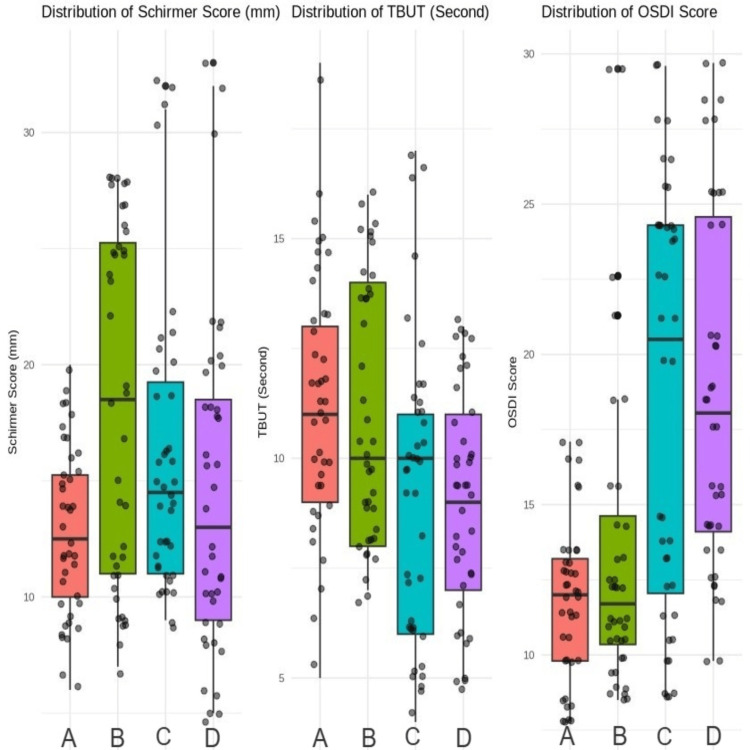
Comparison of Schirmer scores, TBUT, and OSDI scores. (A) Control group. (B) Group 1 were evaluated 0-1 month after BoNT-A injection. (C) Group 2 were evaluated 1-3 months post-injection. (D) Group 3 were evaluated 3-6 months after injection OSDI: Ocular Surface Disease Index; TBUT: tear break-up time; BoNT-A: botulinum toxin-A

OSDI scores revealed differences in symptom severity across the study groups. Groups 2 and 3 exhibited statistically significantly higher mean OSDI scores (18.62±7.04 and 18.82±6.08, respectively) compared to the control group (11.82±2.67; p<0.001). The demographic characteristics and ocular surface parameters for all groups are summarized in Table 1, with comparative analyses shown in Figure 1.

**Table 1:** Comparative analysis of Schirmer scores, TBUT, and OSDI scores across the different study groups OSDI: Ocular Surface Disease Index; TBUT: tear break-up time Statistical analysis was performed using one-way ANOVA to compare the means of Schirmer scores, TBUT, and OSDI scores across the four study groups (group 1, group 2, group 3, and the control group)

	Schirmer score (mm)	TBUT (seconds)	OSDI score
Group 1	18.35±7.63	11.05±2.97	13.58±5.4
Group 2	16.05±6.32	9.18±3.50	18.62±7.04
Group 3	14.55±7.02	9.03±2.54	18.82±6.08
Control group	12.9±3.64	11.35±2.92	11.82±2.67

## Discussion

Our study findings suggest that BoNT-A injections may result in a transient impact on tear film parameters, the most significant changes occurring shortly after the injection. Comprehensive evaluations of tear film characteristics in healthy individuals receiving periorbital injections for cosmetic purposes are lacking in the existing literature.

Therewithal, the utilization of multiple validated diagnostic tools, including the Schirmer test for tear production, TBUT for tear film stability, and OSDI for symptom assessment, provided a comprehensive evaluation of tear film dynamics and ocular surface conditions, enabling a thorough assessment of the multifaceted aspects of ocular surface health.

This study addresses this gap by demonstrating that cosmetic BoNT-A injections can alter tear film characteristics. Specifically, the observed decrease in tear production, as indicated by lower Schirmer test scores, and diminished tear film stability, evidenced by shorter TBUT within the first three months post-injection, indicate that these alterations may contribute to the development of dry eye symptoms in these patients.

These findings suggest that BoNT-A injections can lead to temporary reductions in tear production and impaired tear film stability. The underlying mechanism may be related to the effects of BoNT-A on the orbicularis oculi muscle, which plays a crucial role in the blink reflex and tear production [10,11]. Weakening of the orbicularis oculi can result in a decreased blinking frequency and reduced tear secretion, ultimately leading to an unstable tear film and the development of dry eye symptoms [12]. However, these effects are typically self-limiting and coincide with the known duration of BoNT-A's pharmacological action, suggesting that any associated dry eye symptoms can be effectively managed with temporary ocular surface lubrication until the restoration of normal tear film dynamics.

Previous research has investigated the impact of botulinum toxin on tear drainage and ocular surface parameters. For instance, Sahlin et al. found that limited dose injection produces better results with fewer complications, such as eyelid retraction, in patients with dry eye disease [13]. Similarly, Choi et al. reported an increase in tear meniscus height and improvement in OSDI scores one month after BoNT-A injection in patients with dry eye disease [14]. In contrast, Bayraktar Bilen et al. observed no significant changes in tear film stability or production in patients with blepharospasm and hemifacial spasm [15]. Yao and Malhotra demonstrated a significant decrease in tear production after BoNT-A injections to the upper face and lateral canthal region, with no marked increase in dry eye symptoms [16]. Additionally, Altin Ekin highlighted the therapeutic potential of lacrimal gland botulinum toxin injections in the treatment of epiphora [17]. These findings are consistent with those of the present study's investigation of botulinum toxin in modulating tear film dynamics and addressing related symptom management. While the current study initially shows an increase in Schirmer test results, the subsequent decrease in TBUT and increase in OSDI scores suggest that chemodenervation of the orbicularis oculi muscle may initially increase tears, followed by a more pronounced, time-dependent effect on the lacrimal gland.

The results of this study demonstrate the multifaceted impact of BoNT-A injection on tear production, tear film stability, and ocular surface symptoms. In particular, the decreases in Schirmer values, shorter TBUT durations, and higher OSDI scores observed in the 1-3 month and 3-6 month post-injection groups indicate the time-dependent effects of botulinum toxin on tear dynamics. The effects of BoNT-A on tear film dynamics may not fully manifest within the first month after injection. The modulation of cholinergic signaling and the blockade of parasympathetic innervation of the lacrimal gland can lead to gradual changes in tear production and stability [18]. More pronounced effects therefore typically emerge after the first month. It is crucially important for clinicians to be aware of these temporal variations when evaluating and counseling patients undergoing cosmetic periorbital botulinum toxin injections.

Although the Schirmer test results in groups 2 and 3 were lower than those in group 1, they were not significantly different from those in the control group. This suggests that the impact of BoNT-A injections on tear production, as measured by the Schirmer test, may be more transient and less pronounced in the long term than initially hypothesized. The lack of significant differences between the treatment groups and the control group indicates that the decrease in tear production observed shortly after the injections may be a temporary effect and that the ocular surface may be able to gradually adapt and compensate.

The significant differences in TBUT measurements between groups 2 and 3 and the control group suggest that BoNT-A injections may lead to a sustained reduction in tear film stability. This may be due to prolonged alterations in ocular surface physiology or tear composition that extend beyond the initial post-injection period. The weakening of the orbicularis oculi muscle caused by BoNT-A injection may result in decreased blink frequency and tear secretion, ultimately leading to an unstable tear film and to the development of dry eye symptoms over an extended period.

However, the persistent elevation in OSDI scores in groups 2 and 3 suggests that dry eye symptoms may persist even while tear film parameters improve. This highlights the complexity of ocular surface homeostasis and the potential for persisting symptoms despite objective improvements in tear production and stability. Nevertheless, the impact of BoNT-A injections on the ocular surface appears to be transient, as evidenced by the gradual return to baseline levels in the groups evaluated at later time points [19,20]. This suggests that the ocular surface may have the capacity to adapt and compensate for the initial changes, potentially through the activation of compensatory mechanisms, such as increased tear production by the accessory lacrimal glands.

It is essential to consider these potential side effects when administering BoNT-A, particularly in patients with pre-existing dry eye conditions or those at higher risk of developing dry eye due to factors such as prolonged screen time, environmental exposure, or systemic conditions. Careful patient selection should include a thorough ocular and systemic history, as well as baseline assessments of tear film parameters when feasible. Dosage adjustments should be tailored to minimize the impact on tear film stability, especially in high-risk individuals.

These findings emphasize the importance of extended patient monitoring and proactive management strategies. Practitioners should consider implementing routine tear film assessments during follow-up visits, particularly during months 1-6 post-injection. Additionally, patients should be educated about potential dry eye symptoms and preventive measures, such as the regular use of artificial tears and proper blink exercises, which may help maintain ocular surface comfort during this period.

One limitation of our study is that baseline assessments for dry eye, including tear film and ocular surface parameters, were not conducted prior to the study. However, none of the patients reported any ocular or systemic complaints, which was deemed sufficient to assume baseline ocular health. Additionally, we did not collect data on participants' occupational characteristics or daily screen time exposure. Furthermore, the limitations of this cross-sectional study, which evaluated different groups at various time points post-injection, include the lack of long-term follow-up data beyond six months. Lastly, the relatively small sample size of 80 participants may limit the generalizability of our findings, and future studies with larger cohorts are needed to validate these results.

## Conclusions

This study shows that cosmetic periorbital BoNT-A injections can result in transient alterations in tear film parameters, leading to decreased tear production and tear film instability, as well as increased ocular surface disease symptoms. These changes may develop gradually, highlighting the importance of close monitoring and patient education regarding the potential ocular side effects associated with such injections. Further prospective, longitudinal studies with larger sample sizes and extended follow-up periods are now needed to confirm these findings and provide a more comprehensive understanding of the relationship between cosmetic periorbital botulinum toxin injections and ocular surface dynamics.

## Appendices

**Table 2:** OSDI survey OSDI: Ocular Surface Disease Index; ATM: automated teller machine; TV: television

Questions	All of the time	Most of the time	Half of the time	Some of the time	None of the time
1. Eyes that are sensitive to light?	4	3	2	1	0
2. Eyes that feel gritty?	4	3	2	1	0
3. Painful or sore eyes?	4	3	2	1	0
4. Blurred vision?	4	3	2	1	0
5. Poor vision?	4	3	2	1	0
6. Reading?	4	3	2	1	0
7. Driving at night?	4	3	2	1	0
8. Working with a computer or a bank machine (ATM)?	4	3	2	1	0
9. Watching TV?	4	3	2	1	0
10. Windy conditions?	4	3	2	1	0
11. Places or areas with low humidity (very dry)?	4	3	2	1	0
12. Areas that are air-conditioned?	4	3	2	1	0
